# Tooth Anomalies, Caries, and Gingival Health in Cleft Lip and Palate Patients: A Retrospective Study

**DOI:** 10.3290/j.ohpd.c_2595

**Published:** 2026-04-13

**Authors:** Ege Dogan, Bengisu Eskici, Ece Eden

**Affiliations:** a Ege Dogan Associate Professor, Orthodontic Department, Ege University School of Dentistry, Izmir, Turkey. Conceptualization, methodology, curated data, investigation, prepared original draft, reviewed and edited the manuscript, read and approved the final manuscript.; b Bengisu Eskici Dentist in Private Practice, Izmir, Turkey. Collected data, formal analysis, prepared original draft, read and approved the final manuscript.; c Ece Eden Professor, Pedodontics Department, Ege University School of Dentistry, Izmir, Turkey. Conceptualization, methodology, collected data, prepared original draft, supervised the study, read and approved the final manuscript.

**Keywords:** cleft lip and palate, DMFT index, dmft index, oral health, tooth anomaly.

## Abstract

**Purpose:**

To investigate tooth anomalies, caries status, and gingival health in individuals with different types of cleft lip and palate (CLP).

**Materials and Methods:**

Radiographic and photographic records of 116 patients with CLP were retrospectively examined. Standardized forms were completed in three age-based subgroups (3–5 years, 6–12 years, ≥13 years) and three cleft type groups (right UCLP, left UCLP, BCLP). The DMFT index in permanent teeth, dmft in deciduous teeth, tooth anomalies, and gingival health were recorded. Prevalence of decay and anomalies in the cleft region was evaluated. Kruskal-Wallis and chi-squared tests were used. A significance level of p < 0.05 was considered statistically significant.

**Results:**

The mean DMFT value was 3.96 ± 3.12, and the mean dmft value was 1.18 ± 2.59. In the anterior region, no filled permanent teeth were found in 109 individuals, no supernumerary teeth in 105, and no teeth with shape abnormalities in 97. Healthy gums were observed in 48 patients. The relationship between cleft type and maxillary anterior decay showed statistically significant differences (p < 0.05), and DMFT values also differed statistically significantly among cleft groups (p = 0.001).

**Conclusion:**

Patients not only exhibit poor overall oral hygiene but also a higher prevalence of decay in the cleft region. Adoption of a multidisciplinary approach and collaboration with other healthcare professionals can further enhance overall health and quality of life for these individuals. This is among the first studies in Turkey to simultaneously evaluate anomalies, caries, and gingival health in CLP patients with an a priori power analysis. These findings underline the importance of early, targeted preventive dental strategies in individuals with cleft lip and palate, particularly in the cleft region where caries and dental anomalies are more prevalent. An integrated evaluation of dental anomalies, caries experience, and gingival health may support more effective preventive dentistry and improved long-term oral health outcomes in this high-risk population.

Individuals with cleft lip and palate (CLP) encounter various challenges arising from deviations in normal anatomy. These challenges include difficulties in eating, breathing, hearing, esthetics, phonation, social interactions, and the management of chronic conditions. Consequently, individuals with CLP often find themselves at a disadvantage compared to their peers. Alterations in saliva flow rate, emotional and psychological issues, and negative outcomes associated with oral hygiene further exacerbate these disadvantages.^2,7,8^

The etiology of CLP is multifactorial, involving genetic predisposition, radiation, maternal hypoxia, teratogenic drugs, malnutrition, smoking, alcohol, viral infections, folic acid deficiency or excess, consanguineous marriage, stress, and systemic conditions.^2,7,19^ The worldwide prevalence of CLP is estimated as 1 in 700 to 1 in 1000 births. Syndromes are frequently observed in CLP patients, and the frequency of additional anomalies ranges from 7.5% to 45% in cleft lip and palate cases, and from 13% to 72% in isolated cleft palate.^2,7^

Dental anomalies are among the most frequently observed complications in CLP. Missing or supernumerary teeth, particularly in the cleft area, contribute to malocclusions. Maxillary growth deficiency, exacerbated by scarring after surgical repair, leads to retrusion, crowding, and insufficient vertical development.^7,19^ Clefts in the alveolus affect both deciduous and permanent dentitions, often involving the lateral incisor and canine region, where agenesis, displacement, and morphological deformities are common.^3,8^ Additionally, CLP patients are more prone to caries than their non-cleft peers.

Although many studies have focused on dental anomalies or caries in isolation, few have evaluated dental anomalies, caries status, and gingival health together in the same CLP cohort, and even fewer have analyzed these findings by cleft type. This study was designed to address this gap by retrospectively evaluating these parameters from radiographic and photographic records. The null hypothesis was that tooth anomalies, caries status, and oral hygiene in the cleft region would not differ from those in the non-cleft region of CLP patients.

## MATERIALS AND METHODS 

### Study Design and Ethical Approval

This retrospective study was conducted using radiographic and photographic records obtained from the archives of the Department of Orthodontics, Ege University Faculty of Dentistry, between 2021 and 2022. The study protocol was reviewed and approved by the Ege University Medical Research Ethics Committee (decision No: 23-1.1T/41 date: 26.01.2023). Informed consent was obtained from all individual participants and/or their legal guardians. All data were anonymized prior to analysis.

### Study Population and Eligibility Criteria

The study included patients with unilateral (UCLP) or bilateral (BCLP) cleft lip and palate who had available panoramic radiographs and intraoral photographs in the archive. Patients with isolated cleft palate, syndromic diagnoses, or missing records (photographs or radiographs) were excluded.

Because dental development is strongly age-related, patients were stratified into dentition-based categories:

3–5 years: primary dentition,
6–12 years: mixed dentition, ≥13 years: permanent dentition/adolescents and older.

### Sample Size Calculation (G*Power)

Sample size was estimated using G*Power v3.1. The primary hypothesis was thatdental caries and anomalies differ among CLP types (right UCLP, left UCLP, BCLP). For a chi-squared goodness-of-fit test with three groups, an effect size of Cohen’s w = 0.30 (moderate) was chosen. With α = 0.05 and power (1–β) = 0.90, the required total sample size was ca. 108–119 participants. Our dataset included 116 patients (distribution ≈30/42/44 across cleft types), which provides ≥0.80 power even with unequal group sizes.

The age subgroups were not part of the original sample size calculation and were reported descriptively. Instead, age was included as a covariate in regression analyses to ensure valid comparisons across groups. Importantly, our methodological approach is consistent with recent studies that used formal sample size calculations to ensure adequate power in oral health research, such as Shetty et al,^17^ who designed their large-scale cross-sectional study with a-priori power analysis to secure sufficient sample size.

### Data Collection and Variables

Two calibrated examiners independently reviewed the records using a standardized data collection form. Variables assessed included:

Cleft type (UCLP right, UCLP left, BCLP),
DMFT index (permanent dentition),
dmft index (deciduous dentition),
Congenitally missing teeth (number),
Supernumerary teeth (number),
Morphological anomalies (abnormalities in size/shape),
Gingival health (healthy vs unhealthy).

### Reproducibility (Reliability Analysis)

To ensure reliability, 20% of the sample was randomly re-evaluated after a two-week interval by the same examiner, and independently by a second examiner. Inter- and intra-examiner reliability were assessed using Cohen’s kappa for categorical variables and intraclass correlation coefficients (ICC, two-way mixed, single measures) for count data. Kappa/ICC values ≥0.75 were considered good and ≥0.90 excellent.

### Statistical Analysis

Descriptive statistics were calculated as mean ± SD or median (IQR) for continuous variables, and frequencies (%) for categorical variables. Associations between cleft type and oral health indicators were analyzed using the chi-squared test or Fisher’s exact test where appropriate. For quantitative indices (DMFT/dmft), group comparisons were made using the Kruskal-Wallis test. Additionally, logistic regression and Poisson/negative binomial regression were performed, adjusting for age and sex. Multiple comparisons were corrected using the Benjamini–Hochberg false discovery rate method. Statistical significance was set at p < 0.05.

Although age groups are presented for descriptive purposes, age was analyzed as a continuous variable in regression models to preserve statistical power and to avoid information loss associated with arbitrary categorization.

## RESULTS 

A total of 116 children were included in the study, of whom 67.2% (n = 78) were male. Distribution by cleft type was: right unilateral CLP (n = 30, 19.0%), left unilateral CLP (n = 42, 36.2%), and bilateral CLP (n = 44, 37.9%). Age distribution was 3–5 years (n = 14), 6–12 years (n = 86), and ≥13 years (n = 16).

### General Oral Findings

The frequencies of DMFT, dmft, tooth anomalies, supernumerary teeth, and congenitally missing teeth are presented in Table 1. Overall, 41.4% (n = 48) of the patients had clinically healthy gingiva, while the majority showed gingival pathology.

**Table 1:** Frequencies of oral findings in patients with cleft lip and palate (n = 116)

Score	DMFT	dmft	Tooth anomaly	Supernumerary tooth	Congenital missing tooth
0	15	82	97	105	19
1	12	8	14	6	42
2	16	7	4	5	40
3	24	4	1	–	8
4	4	4	–	–	5
5	11	3	–	–	2
6	9	2	–	–	7
7	6	1	–	–	–
8	8	2	–	–	–
9	6	1	–	–	–
10	3	–	–	–	–
13	–	1	–	–	–
14	2	–	–	–	–
15	–	1	–	–	–
Total	116	116	116	116	116


The distribution of decayed, missing, filled, supernumerary, and congenitally missing teeth in the maxillary anterior region is shown in Table 2. No filled permanent teeth were found in 109 individuals (94.0%), while supernumerary teeth were absent in 105 (90.5%). Congenitally missing teeth in the anterior maxilla were observed in 21 patients.

**Table 2:** Distribution of dental findings in the maxillary anterior region among patients with CLP

Score	D	d	M	m	F	f	Supernumerary tooth	Missing tooth
0	64	109	116	116	109	116	105	21
1	28	6	–	–	4	–	7	43
2	17	1	–	–	3	–	4	42
3	4	–	–	–	–	–	–	4
4	3	–	–	–	–	–	–	6


### Association with Cleft Type

The Kruskal-Wallis analysis revealed statistically significant associations between cleft type and several oral health parameters. Specifically, the number of decayed permanent teeth in the anterior region differed stastically significantly between cleft groups (χ^2^ = 8.124, df = 3, p = 0.044) and DMFT scores were also significantly different (χ^2^ = 17.575, df = 3, p = 0.001) (Table 3). In contrast, no stastically significant differences were found for dmft values (p = 0.093) or morphological anomalies in the anterior region (p = 0.860).

**Table 3:** Kruskal-Wallis test for the relationship between cleft type and maxillary anterior DMFT/dmft

Variable	Χ^2^	df	p-value
Supernumerary teeth	2.492	3	0.477
Supernumerary teeth (maxilla)	2.438	3	0.487
Morphological deformities (anterior)	0.754	3	0.860
Filled deciduous teeth (anterior)	0.000	3	1.000
Filled permanent teeth (anterior)	0.918	3	0.821
Decay deciduous teeth (anterior)	0.690	3	0.875
Decay permanent teeth (anterior)	8.124	3	0.044*
DMFT	17.575	3	0.001*
dmft	6.416	3	0.093
* Statistically significant at p < 0.05.			

In addition, congenital tooth deficiency was strongly associated with cleft type. Both the total number of congenitally missing teeth (χ^2^ = 35.871, df = 3, p < 0.001) and the number of congenitally missing teeth in the maxilla (χ^2^ = 39.840, df = 3, p < 0.001) differed stastically significantly across cleft types (Table 4).

**Table 4:** Relationship between maxillary decay, tooth deficiency, and CLP type (Kruskal-Wallis test)

Variable	χ^2^	df	p-value
Maxillary DMF	7.087	3	0.069
Maxillary dmf	1.250	3	0.741
Congenitally missing teeth	35.871	3	<0.001*
Congenitally missing teeth (maxilla)	39.840	3	<0.001*
*Statistically significant at p < 0.05.			

### Anterior Caries Distribution

The distribution of caries in the maxillary anterior region is summarized in Table 5 (permanent teeth) and Table 6 (deciduous teeth). More than half of the patients (55.2%) showed no permanent anterior caries, while 44.8% had at least one carious permanent tooth. In the deciduous anterior dentition, 94.0% of the patients were caries-free, with only 6.0% presenting one or two carious teeth.

**Table 5:** Number of children with caries in permanent teeth in the maxillary anterior region

Score	n	%
0	64	55.2
1	28	24.1
2	17	14.7
3	4	3.4
4	3	2.6
Total	116	100.0


**Table 6:** Number of children with caries in deciduous teeth in the maxillary anterior region

Score	n	%
0	109	94.0
1	6	5.2
2	1	0.9
Total	116	100.0


### Data Availability

The datasets used and/or analyzed during the current study are available from the corresponding author on reasonable request.

## DISCUSSION 

CLP is commonly associated with dental anomalies involving number, size, shape, structure, position, and eruption, affecting both dentitions. Dental anomalies, including hypodontia, supernumerary teeth, hypoplasia, and abnormalities in tooth size and shape have also been associated with CLP. The prevalence of hypodontia in CLP patients has been reported to range from 31.6% to 77%. Additionally, the prevalence of hypodontia increases with the severity of the cleft. Apart from the commonly missing lateral incisor, other frequently involved teeth are the maxillary and mandibular second premolars with the maxillary second premolar being the most frequently absent tooth. It has been reported that the prevalence of a supernumerary lateral incisor in CLP patients ranges from 5.1% to 22.1%. Other dental anomalies associated with CLP include abnormalities in the shape and size of permanent teeth, especially in the anterior maxillary region.^3,4,8,14,15^ According to the results of this study, patients with CLP showed more dental anomalies.

Furthermore, differences in the associations of dental anomalies with cleft type were observed. All dental anomalies were seen in higher proportions among patients with complete clefts compared to patients with incomplete clefts. The absence of fusion between the maxillary and medial nasal processes, resulting in CL/P, may contribute to various anomalies affecting the lateral incisor. This could explain the frequent absence of lateral incisors or their distal or mesial location with respect to the cleft, as well as the presence of supernumerary teeth in the same region. The presence of supernumerary teeth in this region is attributed to the susceptibility of the tooth buds of the permanent lateral incisors to modification or division, possibly being divided by the cleft.^10,15,19^

According to the study by Răducanu et al,^15^ dental anomalies are more prevalent in patients with CLP around the cleft area and occur with a higher frequency in individuals with oral clefts than in the general population. That study demonstrated that the proportion of patients with shape anomalies in the CLP sample was statistically significantly higher than in the control group. Dilaceration and peg-shaped teeth were the most common tooth shape anomalies in the areas affected by CLP, while a supplementary cusp was the most frequently encountered morphological abnormality in the control group.

According to the review by Qadeer et al,^14^ agenesis of the lateral incisor is the most common dental anomaly in CLP, among the various abnormalities in tooth form, shape, number of teeth, structural disturbances, and eruption sequences that were reported. Jamilian et al^11^ stated that the vast majority of CLP patients had at least one dental anomaly, and most of these anomalies were observed on the side of the cleft. However, no association could be found between the type of cleft and dental anomalies. The study showed that dental anomalies are very common in all types of CLP patients, and there are no specific associations between the types of cleft and tooth anomalies, including incisors and laterals rotation, opacity, hypoplasia, and peg-shaped laterals. Akcam et al^1^ reported that a significant proportion (96.7%) of individuals with a cleft had at least one dental anomaly.

Pradhan et al^13^ conducted a descriptive cross-sectional study utilizing 208 radiographs collected from cleft lip and palate patients. Dental anomalies were highly prevalent, with at least one anomaly present in 188 (90.4%) of patients, with males (57.4%) showing a higher incidence than females (42.6%). The most common anomaly was dental agenesis, observed in 161 (77.9%) cases. The prevalence of positional anomalies, morphological anomalies, and supernumerary teeth were found to be 54 (26%), 33 (15.9%), and 20 (10%), respectively. Among all missing teeth, the lateral incisor exhibited the highest incidence of agenesis at 65.2%.

In a systematic review examining studies on the improvement of oral hygiene in individuals with CLP, it was reported that caries and oral hygiene are worse in individuals with CLP.^1,11,13^ In our study, the average DMFT value for individuals with CLP was 3.9569, and the dmft value was 1.1810. In a similar age group in a study by Pisek et al,^12^ the DMFT value ranged from 0.82 to 1.23, and the dmft value ranged from 0.66 to 1.38, indicating lower values than in our study. Conversely, a group of patients of the same age from Hazza’a et al.^9^ exhibited higher DMFT and dmft values, ranging from 4.58 to 5.37 and 4.28 to 4.19, respectively, than in our study.

In the oral and dental health profile research conducted in Turkey, the decay prevalence for healthy 5-year-old children was reported as 64.4%, with a dmft value of 3.64±4.04. For 12-year-olds, the prevalence was 46.6%, and the DMFT was 1.57±2.16. This indicates that tooth decay is a common issue in the deciduous dentition of children in Turkey, and the presence of CLP can exacerbate this condition.^18^ The variation in reported decay prevalence values among countries may be associated with community awareness levels and socioeconomic differences between examined groups. However, consistently high values of DMFT and dmft in all studies, particularly when compared to the averages of healthy groups, emphasize that individuals with CLP are at a significant disadvantage.^3,5,6,16^ This disadvantage appears to be independent of cultural influences.

An overarching point in the studies is that patients generally have poor gingival health.^5,9,12^ This has also been confirmed in our study. According to our study findings, CLP negatively affects gingival health as well. In a study conducted by Hazza’a et al,^9^ the amount of decay in women was found to be higher than in men, while in our study group, no statistically significant relationship was found between gender and the amount of decay.

Importantly, our study design incorporated an a-priori power analysis to ensure adequate sample size, consistent with recommendations in recent oral health research. A large-scale cross-sectional investigation by Shetty et al^17^ similarly emphasized the critical role of proper sample size calculation in avoiding underpowered studies and demonstrated how unmet dental needs significantly influence oral health–related quality of life. The methodological consistency between our study and that report supports the robustness of our findings.

Individuals with CLP constitute a disadvantaged group in terms of oral hygiene due to their physical anomalies. The results obtained in the study confirm this assertion. In our examined group, it was observed that the number of permanent decayed teeth in the anterior region, the DMFT value, the number of congenitally missing teeth, and the number of congenitally missing teeth in the maxilla are associated with the type of CLP. Notably, these values increase as they approach the cleft region.^5,11,15,16^

An important strength of the present study is the integrated evaluation of dental anomalies, caries experience, and gingival health within the same cleft lip and palate cohort. While previous studies have often focused on a single oral health parameter, the combined assessment used in this study provides a more holistic understanding of oral health status in relation to cleft type and is particularly relevant for preventive dentistry and multidisciplinary clinical management.

## CONCLUSION 

The null hypothesis that tooth anomalies, caries status, and oral hygiene in the cleft region are not different from those in the non-cleft region of CLP patients has been rejected. Individuals with CLP experience challenges in nutrition, respiration, hearing, esthetics, phonation, and social interactions, and may encounter chronic health issues due to anatomical deviations. These challenges place them at a disadvantage compared with their peers of the same age.

The novelty of this study lies in the comprehensive evaluation of dental anomalies, DMFT/dmft values, and gingival health in the same CLP cohort, analyzed in relation to cleft type. Unlike previous reports that focused on isolated findings, our results provide an integrated perspective, supported by an a priori power analysis ensuring sufficient sample size and robust statistical validity.

Our findings indicate that patients with CLP not only exhibit poor overall oral hygiene but also have a higher prevalence of dental caries and anomalies in the cleft region. Anatomical differences appear to hinder effective cleaning of these areas, contributing to the observed outcomes. Therefore, dentists should provide detailed guidance to CLP patients, especially at younger ages, and educate their parents on proper oral hygiene habits. Instilling preventive oral care practices early is crucial for maintaining dental health and reducing disease burden. Finally, a multidisciplinary approach, incorporating pediatric dentistry, orthodontics, surgery, and speech therapy, will further improve oral health outcomes and overall quality of life for individuals with CLP.
